# Horizontal gene transfer in human-associated microorganisms inferred by phylogenetic reconstruction and reconciliation

**DOI:** 10.1038/s41598-019-42227-5

**Published:** 2019-04-11

**Authors:** Hyeonsoo Jeong, Bushra Arif, Gustavo Caetano-Anollés, Kyung Mo Kim, Arshan Nasir

**Affiliations:** 1 Department of Animal Sciences, University of Illinois at Urbana-Champaign, Urbana, IL USA; 2 School of Biological Sciences, Georgia Institute of Technology, Atlanta, GA United States; 3 Department of Biosciences, COMSATS University Islamabad, Park Road, Tarlai Kalan, Islamabad, Pakistan; 4 Department of Crop Sciences, University of Illinois at Urbana-Champaign, Urbana, IL USA; 5 Division of Polar Life Sciences, Korea Polar Research Institute, Incheon, Republic of Korea

## Abstract

Horizontal gene transfer (HGT) is widespread in the evolution of prokaryotes, especially those associated with the human body. Here, we implemented large-scale gene-species phylogenetic tree reconstructions and reconciliations to identify putative HGT-derived genes in the reference genomes of microbiota isolated from six major human body sites by the NIH Human Microbiome Project. Comparisons with a control group representing microbial genomes from diverse natural environments indicated that HGT activity increased significantly in the genomes of human microbiota, which is confirmatory of previous findings. Roughly, more than half of total genes in the genomes of human-associated microbiota were transferred (donated or received) by HGT. Up to 60% of the detected HGTs occurred either prior to the colonization of the human body or involved bacteria residing in different body sites. The latter could suggest ‘genetic crosstalk’ and movement of bacterial genes within the human body via hitherto poorly understood mechanisms. We also observed that HGT activity increased significantly among closely-related microorganisms and especially when they were united by physical proximity, suggesting that the ‘phylogenetic effect’ can significantly boost HGT activity. Finally, we identified several core and widespread genes least influenced by HGT that could become useful markers for building robust ‘trees of life’ and address several outstanding technical challenges to improve the phylogeny-based genome-wide HGT detection method for future applications.

## Introduction

Horizontal gene transfer (HGT) of antibiotic resistance genes, virulence factors, toxins, and genes involved in stress response and metabolism are pervasive among prokaryotes, especially bacteria^1^. HGT is even more widespread in the human-associated microorganisms^2–4^ due to the close physical proximity and increased cell-to-cell contact within the human body (e.g. biofilm formation in the oral cavity and the gastrointestinal tract). The phylogenetic structure of microbial communities can further increase HGT likelihood^5^ since closely-related microorganisms that share similar mobilomes^6^ are expected to colonize similar habitats. Proximity provides opportunities for genetic exchange occurring via plasmid (conjugation) and phage mediated gene transfer mechanisms (transduction) and uptake of exogenous DNA (transformation) from the environment. How the short-range (i.e. HGTs within a body site) and long-range gene transfers (i.e. HGTs between body sites) occur within the human body in the context of ecological, temporal, spatial, and phylogenetic structure of microbial communities are therefore vital questions to understand how the human microbiota establishes biochemical and genetic networks responsible for maintaining host physiology and health^2^.

Accurate detection and classification of ancient and recent HGT events remains a major computational and conceptual challenge^7,8^. Historically, sequence similarity and composition-based statistics such as GC content, oligonucleotide frequency and codon usage biases^9–11^ have been used to discriminate horizontal from vertical evolution. However, composition-based methods work poorly for ancient gene transfers^12,13^ and tend to give contrasting results when the methods are changed^14^. For example, a recent study implemented a BLAST-based method to detect highly similar nucleotide regions (>99% identity in blocks of >500 bp) across distantly related genomes (<97% 16S ribosomal RNA similarity) as evidence of HGT^2^. The authors discovered that long-range gene transfers occurred more frequently within the human body than among the genomes of human-associated and non-human environments suggesting ecology was the major driver behind increasing HGT^2^. While their method was powerful, it was limited to the detection of recent HGT events since statistically detectable sequence similarity fades over evolutionary time^15^. To better detect ancient HGT events across diverse prokaryotic species, phylogeny-based approaches of gene-species tree reconstruction and reconciliation can provide deeper resolution^16^.

**Figure 1:**
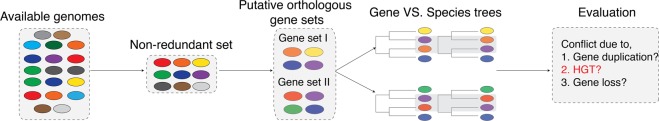
HGT detection workflow. From a large pool of available completely sequenced genomes, non-redundant genomes are filtered and selected for downstream analysis. Putative orthologous gene sets and corresponding reference species trees are then reconstructed based on different criteria (e.g. NJ, ML, and other approaches^16^). Gene sets are called ‘putative’ orthologs as they are subjected to downstream tests for HGT participation. Each gene-species tree pair is evaluated for topological incongruence (see the dark shaded area in trees). Tree conflicts can arise from any of the following gene family evolution events: (i) duplication, (ii) HGT, and (iii), gene loss, commonly known as the duplication-transfer-loss (DTL) problem^20^. Out of the most parsimonious reconciliation (in terms of total cost of gene family evolution events)^20^, conflicts arising from transfer are stored for further analysis.

Here, we performed >80,000 gene and species phylogenetic tree reconstructions and reconciliations to detect “candidate” HGT events in 1,059 reference prokaryotic genomes sequenced from six major body sites of “healthy” human adults sampled by the NIH Human Microbiome Project (HMP)^17–19^. We used a modified version of the *HGTree* pipeline, a phylogeny-based HGT detection method previously developed by Jeong *et al*.^16^. *HGTree* is based on an explicit evolutionary method implementing a combination of parsimony^20^, neighbor-joining^21^, and maximum likelihood (ML)^22^ approaches to reconstruct and compare topologies of gene trees against corresponding 16S ribosomal RNA (rRNA) species (reference) trees (Fig. 1). Gene-species tree reconciliations are evaluated under a parsimony framework^20^ where a cost is assigned to each of the four possible events describing gene family evolution (i.e. speciation, duplication, horizontal transfer, and loss). The objective is to find the most-parsimonious gene-species tree reconciliation that minimizes the total cost and to identify nodes labeled by transfers on the gene trees (see Methods)^20^. The detected “candidate” HGT events along with designations of donor and recipient genomes are stored in the online *HGTree* database (Fig. 1). The *HGTree* database (available at http://hgtree.snu.ac.kr/) therefore provides quick access to pre-calculated HGT events in 2,472 genomes of prokaryotic species isolated from diverse natural habitats (hereafter, *HGTree-genomes*, Supplementary Table S1) and can easily be applied to user-provided genomic datasets (e.g. *HMP-genomes*, Supplementary Table S2).

Implementing the slightly-modified version of the HGTree pipeline to *HMP-genomes* (see Methods), we observed that HGT activity increased significantly for each gene horizontally exchanged by the *HMP-genomes* relative to *HGTree-genomes* and that more than half of the total genes in *HMP-genomes* were transferred (donated or received) by HGT. We found that roughly 40% of the total detected HGT events occurred among microorganisms sharing the same niche or body site (i.e. due to ecological similarity and physical proximity) and HGT activity was strongly influenced by the phylogenetic diversity of the ecosystem. The remaining HGT events (~60%) either indicated transfer of DNA from one body site to another through hitherto poorly understood mechanisms or predated microbial colonization of the human body. We also identified several core genes that were widely shared by microorganisms and evaluated their HGT sensitivity (tendency to participate in HGT). We discovered that several of the well-known phylogenetic markers (e.g. ribosomal proteins, transcription factors) were highly sensitive to HGT questioning their broad use in concatenation-based phylogenies. Finally, we highlight several challenges in the large-scale implementation of phylogeny-based HGT detection methods and suggest several strategies to improve their utility in continuously emerging microbiome and (meta)-genome datasets.

## Results

### Possible multi-residence and contamination of microbial strains and species across human body sites

**Figure 2:**
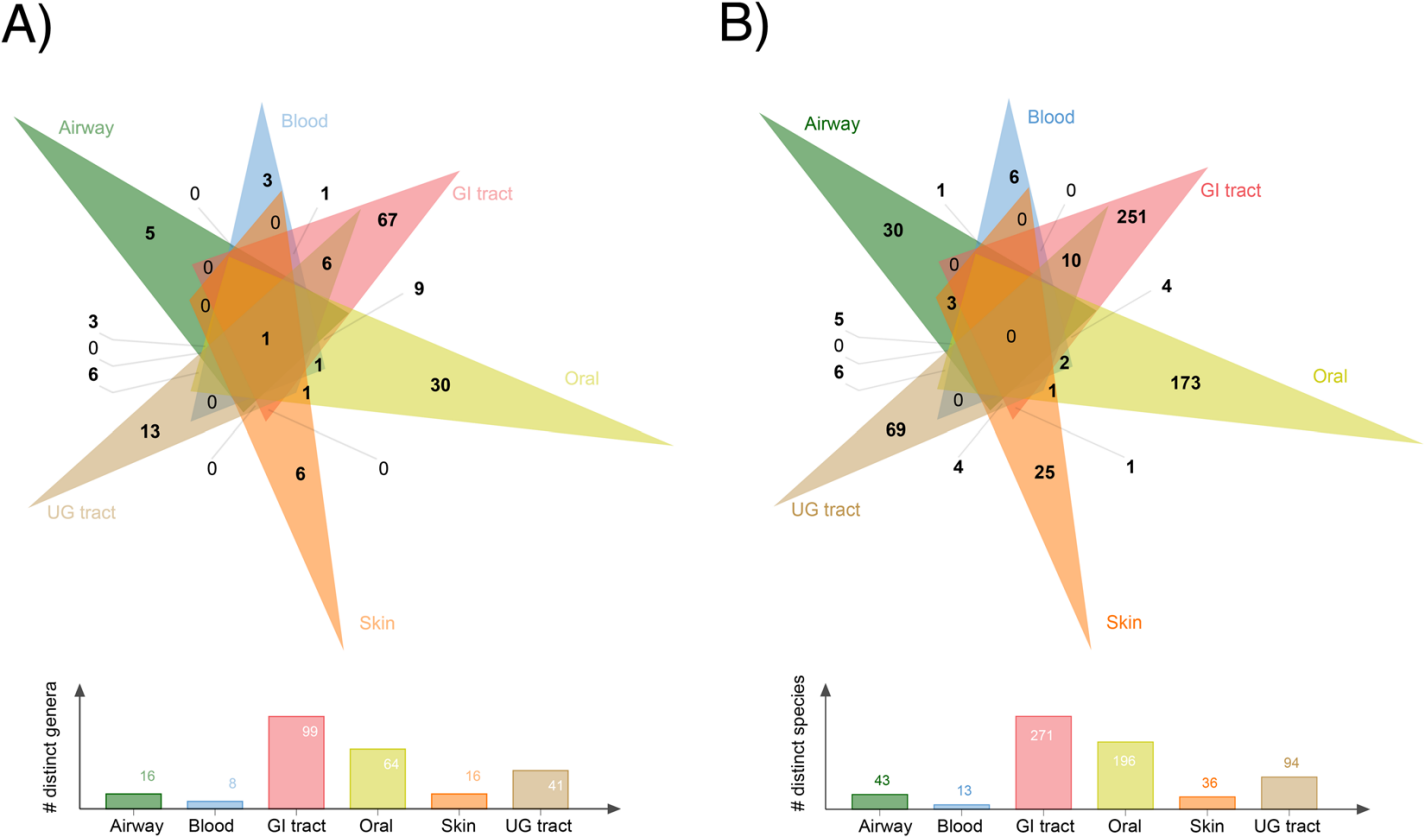
Genus and species composition of studied body sites. Six-way Venn diagrams describe the genus (**A**) and species (**B**) composition of each body site and its combinations with other body sites in the *HMP-genomes* dataset. Histograms below give the total count of total genera and species present in each body site. Genome names having distinct suffixes following “sp.” were treated as different species (Supplementary Table S2). Diagram generated using online version of the jvenn program^80^ available from (http://jvenn.toulouse.inra.fr/app/index.html).

Accurate classification of long-range or *inter-niche* gene transfers (i.e. transfers involving bacteria residing in different body sites) can be biased by the possible multi-residence of bacterial strains and species across the human body sites or contamination resulting from laboratory protocols^23^. Since each sequenced microbial genome corresponds to a microbial strain and a collection of closely-related genomes (typically >95% average nucleotide identity) correspond to a microbial species^24^, we filtered redundant strains/genomes from the HMP dataset and confirmed that none of the remaining 1,059 *HMP-genomes* were associated with more than one body site at the strain level. The *HMP-genomes* corresponded to 152 distinct genera and 591 distinct species that were sequenced from six major human body sites, the gastrointestinal (GI) tract (452 genomes), oral cavity (244), airways (49), skin (123), urogenital (UG) tract (146) and blood (45) (Supplementary Table S2). Out of the total, 124 (82%) genera and 554 (93.7%) species were associated with distinct body sites suggesting significant diversity in microbial colonization across the human body and very little overlap, especially at the species level (Fig. 2). To further verify mono-residence of genomes, we calculated an average nucleotide identity (ANI)^24^ score for each genome pair in the *HMP-genomes* dataset. Only 8 and 918 out of 408,385 possible genome pairs residing in different body sites matched with >99.9% and >95% identity for strains and species (see highlighted rows in Supplementary Tables S3 and S4), respectively, suggesting that multi-residence of species or strains would be a negligible issue in our calculations of gene transfers. The GI tract harbored the maximum number of unique genera (*n* = 67) and species (251). Three distinct genera and six distinct species were also detected in blood (Fig. 2) and only one out of 45 blood-associated microbial genomes (2.2%) matched the list of 93 possibly contaminant genera from Salter *et al*.^23^ (Supplementary Table S5), justifying the inclusion of blood as a valid body site in the analysis. For other body sites, these values ranged between 5–11% except skin for which 90 out of 123 (73%) genomes matched possibly contaminant genera previously reported^23^ (Supplementary Table S5). Skin microbiota, however, comprised of 16 distinct genera and 36 species (Fig. 2) but 71 out of the 90 matching genomes belonged to various strains of a single species, *Propionibacterium acnes*, which is a dominant resident of human skin microbiota and a potential contaminant in clinical samples^25^. Since our study focused on “healthy” individuals as identified by the exhaustive HMP screening and isolation protocols and because *Propionibacterium* represented only one out of a total of 16 skin genera involved in HGT analysis, we are confident that large-scale contamination does not exist in the dataset. Even matching names can only be suspected contaminants since the Salter *et al*.^23^ list even includes *Escherichia*, which is a core member of the human gut microbiota.

### HGT activity is appreciably higher in the genomes of human microbiota relative to environmental microorganisms

All *HMP-genomes* were processed simultaneously for the construction of ‘putative’ orthologous gene sets (putative since we test for their HGT sensitivity participation in downstream analysis). We generated a total of 81,357 gene sets, out of which 26,298 (32%) did not produce detectable gene-species tree conflict evidence of HGT in downstream analysis and were thus tentatively termed *HGT-free* genes. Of the remaining 55,059 (68%) *HGT-genes*, 8,976 (16%) gene sets included member genomes unique to a body site (i.e. *unique* genes) and 46,083 (84%) gene sets included genomes residing in two or more than two body sites (i.e. *mixed* genes). A total of 511,330 HGT events were detected post phylogenetic tree reconstruction and reconciliation of both *unique* (*n* = 17,665 HGT events) and *mixed* (*n* = 493,665) gene sets in the 1,059 *HMP-genomes*. For reference, a total of 660,894 pre-calculated HGT events were retrieved from 93,028 *HGT-gene* sets (out of a total of 154,805 gene sets) in 2,472 *HGTree-genomes* (Table 1). Therefore, *HMP-genomes* encoded roughly 1.38-times greater number of *HGT-genes* per genome relative to *HGTree-genomes* (55,059/1,059 = 51.99 vs. 93,028/2,472 = 37.63) and 1.3-times greater HGT events per each *HGT-gene* (511,330/55,059 = 9.28 vs. 660,894/93,028 = 7.10) relative to *HGTree-genomes*. However, the numbers of *HGT-genes* in genomes and *HGT-events* detected on each gene tree also depend on genome sizes (e.g. larger genomes can have more HGT-derived genes relative to small-sized genomes), size of gene families (e.g. larger gene sets can be expected to have greater number of HGTs), and taxonomic diversity of the gene sets (e.g. gene sets including many closely-related genomes may exchange more genes), as previously discussed^16,26^. These natural sources of variations exist in most biological databases but are expected to have negligible effects when sample sizes are large such as those of the present study. Therefore, we estimate that HGT activity (proportionally) increased by up to 30% in the genomes of human-associated microorganisms relative to environmental microorganisms, which also supports previous findings of significantly higher HGT activity in human-associated microorganisms relative to environmental microorganisms^2^.

**Table 1:** Composition of HMP and *HGTree* derived datasets used in this study. *HGT-genes* produced detectable conflict during gene and species tree reconciliation and this conflict was evaluated to be a result of HGT rather than gene duplication and loss (two other competing scenarios for gene family evolution), as evaluated by RANGER-DTL (ver. 1.0) software^20^.

Dataset	*a*	*b*	*c*	*d*	*e*	*f*	# Gene sets	*# HGT-genes*	# HGT events
*HMP-genomes*	1,059	8	152	591	2	1,057	81,357	55,059	511,330
*HGTree-genomes*	2,472	41	699	1,321	156	2,316	154,805	93,028	660,894

*a,* number of genomes,

*b,* number of distinct phyla,

*c,* number of distinct genera,

*d,* number of distinct species,

*e,* number of archaeal genomes,

*f,* number of bacterial genomes.

### Classification and timing of *intra-* and *inter-niche* gene transfers

**Figure 3:**
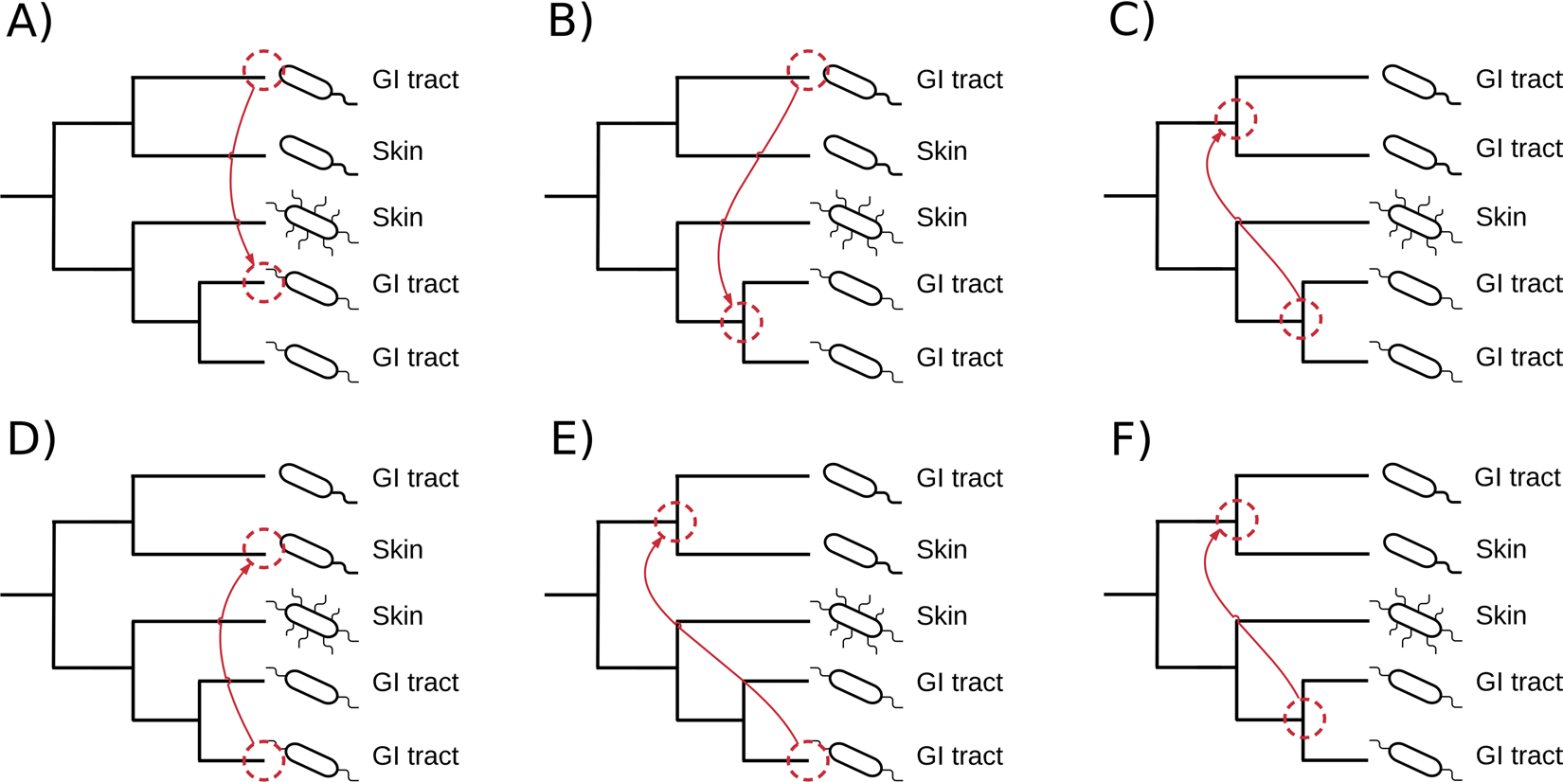
The many faces of HGT. The *intra-niche* HGT events occur between genomes occupying the same body site either in *unique* or *mixed* phylogenetic trees and involve either *one-to-one* (**A**), *one-to-many* (or *many-to-one*) (**B**), or *many-to-many* gene transfers (**C**). The *inter-niche* HGT events occur among genomes occupying different body sites and involve *one-to-one* (**D**), *one-to-many* (or *many-to-one*) (**E**), or *many-to-many* (**F**) transfers, as illustrated on the trees.

**Figure 4:**
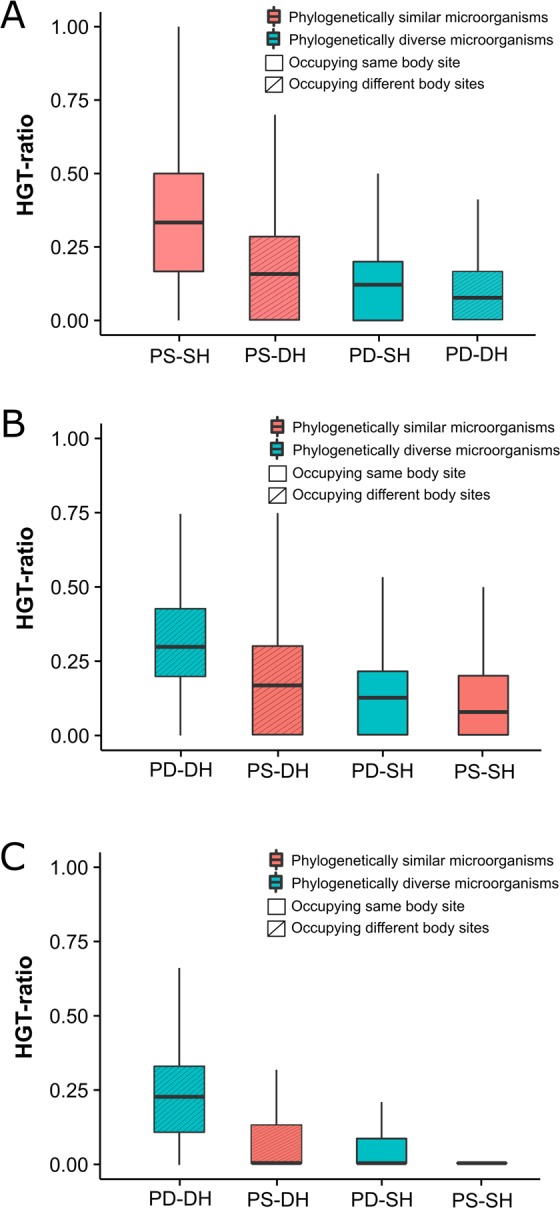
Evaluation of phylogenetic versus spatial effect. HGT ratio represents the total number of *one-to-one* (**A**), *one-to-many* (**B**), and *many-to-many* (**C**) HGT events detected on a gene tree divided by the total number of HGT events (i.e. the sum of *one-to-one*, *one-to-many*, and *many-to-many*) detected on that gene tree. Phylogenetically similar microorganisms (PS) belong to the same genus. Phylogenetically diverse microorganisms (PD) belong to different genera. Similar habitat (SH) implies microorganisms harboring the same body site or niche. Different habitats (DH) imply microorganisms residing in different body sites or niches. See Supplementary Table S6 for *P*-values, pairwise Mann–Whitney *U* test.

HGT events detected on phylogenetic trees were broadly classified into *intra-niche* and *inter-niche* HGTs based on the associations of *HMP-genomes* to human body sites (Fig. 3). The *intra-niche* HGTs involved *one-to-one* (Fig. 3A), *one-to-many* (Fig. 3B), and *many-to-many* (Fig. 3C) gene transfers among genomes that occupied the same body site (e.g. the GI tract). In turn, *inter-niche* HGTs involved *one-to-one* (Fig. 3D), *one-to-many* (Fig. 3E), and *many-to-many* (Fig. 3F) gene transfers among genomes occupying different body sites (e.g. between bacteria residing in the GI tract and the oral cavity). The *intra-niche* HGTs were detected both in the *unique* and *mixed* gene trees, while, *inter-niche* HGTs, by definition, were restricted to *mixed* gene trees. Out of the total 511,330 detected HGT events in *HMP-genomes*, 206,980 (40%) were recognized as *intra-niche* transfers while 304,350 (60%) involved *inter-niche* HGTs involving genomes dispersed on different body sites (see Table 2 for breakdown of detected HGT events by body sites). In general, microorganisms sharing the same niche and spatial proximity have more chances of genetic communication either by establishing direct cell-to-cell contact (e.g. biofilm formation) or via phage/plasmid mediated gene transfers (i.e. the ecological effect)^27,28^. This is especially true if the genetic exchange occurs between closely-related microorganisms. In turn, *inter-niche* transfers indicate either long-range gene transfers that transcend spatial boundaries or ancient gene transfers that occurred prior to the colonization of the human body and have accumulated over time thus explaining their larger number^29^. Because *one-to-one* HGT events are direct gene transfers between genomes residing in either the same or different body sites, they are likely to be more recent in evolution compared to *one-to-many* and *many-to-many* transfers that involve many genomes dispersed across different body sites. Therefore, we calculated an HGT-ratio separately for *one-to-one*, *one-to-many*, and *many-to-many* HGT events, defined by the number of detected events of one category (e.g. *one-to-one*) divided by all events on that gene tree, for *HMP-genomes* belonging to same and different genera (i.e. the phylogenetic effect) separately for *intra-niche* and *inter-niche* HGTs (i.e. the spatial/ecological effect) (Fig. 4, Supplementary Table S6 for *P*-values, pairwise Mann–Whitney *U* test).

**Table 2:** HGT events detected in the microbial genomes of each body site.

Body sites	# Genomes	# total genera	# total phyla	*a*	*b*	*c*	*d*	*e*
Airways	49	16	4	34	3708	3742	98559	26.34
Blood	45	8	3	6	465	471	50360	106.92
GI tract	452	99	7	16301	139668	155969	219505	1.41
Oral	244	64	8	1080	37201	38281	216387	5.65
Skin	123	16	4	38	2843	2881	91880	31.89
UG tract	146	41	7	206	5366	5572	157006	28.18

*a,* number of *intra-niche* gene transfers detected in *unique* gene sets,

*b,* number of *intra-niche* gene transfers detected in *mixed* gene sets,

*c, *sum of *a* and *b*,

*d, *number of *inter-niche* gene transfers detected in *mixed* gene sets,

*e, ratio *(*d*/*c*).

**Figure 5:**
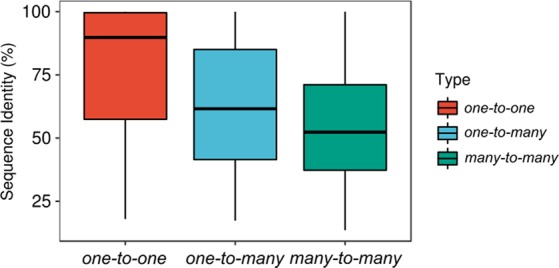
Timing of detected HGT events. Protein sequence identity decreases in the order, *one-to-one*, *one-to-many*, and *many-to-many* for each pair of sequences involved in gene transfer. All comparisons were statistically significant (*P* < 2.2e-16 for all comparisons, Mann–Whitney *U* test).

In *one-to-one* events, the median HGT ratios decreased in the following order: phylogenetically similar microorganisms occupying the same habitat (median = 0.33), phylogenetically similar microorganisms occupying different habitats (0.16), phylogenetically diverse microorganisms occupying same habitats (0.12), and phylogenetically diverse microorganisms occupying different habitats (0.08) (Fig. 4A). Thus, the median HGT ratio decreased from 0.33 to 0.16 (~50% reduction) when microorganisms belonging to the same taxonomy were spatially separated. However, 0.16 was still the second highest median, exceeding the median HGT ratios of microorganisms belonging to different taxonomies but occupying either the same (0.12, an additional 25% reduction) or different habitats (0.08, an additional 25% reduction). Therefore, human-associated microorganisms preferentially exchanged genes with members belonging to the same taxonomy. However, HGT ratios almost doubled when phylogenetically similar microorganisms were also united by spatial proximity, as expected^2^. Since closely-related microorganisms are expected to harbor a similar mobilome^6^, the phylogenetic diversity of environmental samples can be influential in increasing HGT. In turn, median HGT ratios for *one-to-many* and *many-to-many* gene transfers exhibited a trend opposite to that of *one-to-one* HGT events and phylogenetically diverse microorganisms occupying diverse habitats had the highest median HGT ratios (Figs. 4B and 4C). Tentatively, therefore, *one-to-one* events likely include relatively more direct and recent genetic exchanges among prokaryotic species that are united by taxonomy (and physical proximity), while, *one-to-many* and *many-to-many* HGTs likely include more ancient gene transfers involving ancestors of prokaryotic species that likely predated species divergence and colonization of the human body. This was also supported by decreasing mean protein sequence identity between pairs of sequences corresponding to *one-to-one*, *one-to-many*, and *many-to-many* events suggesting these events corresponded to evolutionary time (Fig. 5, *P* < 2.2e-16 for all comparisons, Mann–Whitney *U* test). Note however that this may only represent the generic trend and not the rule and many *one-to-one* events could still be ancient and many *one-to-many* and *many-to-many* events could still be recent.

### The “gut” and “blood” microbiota: The hub and conduit of genetic crosstalk

**Figure 6:**
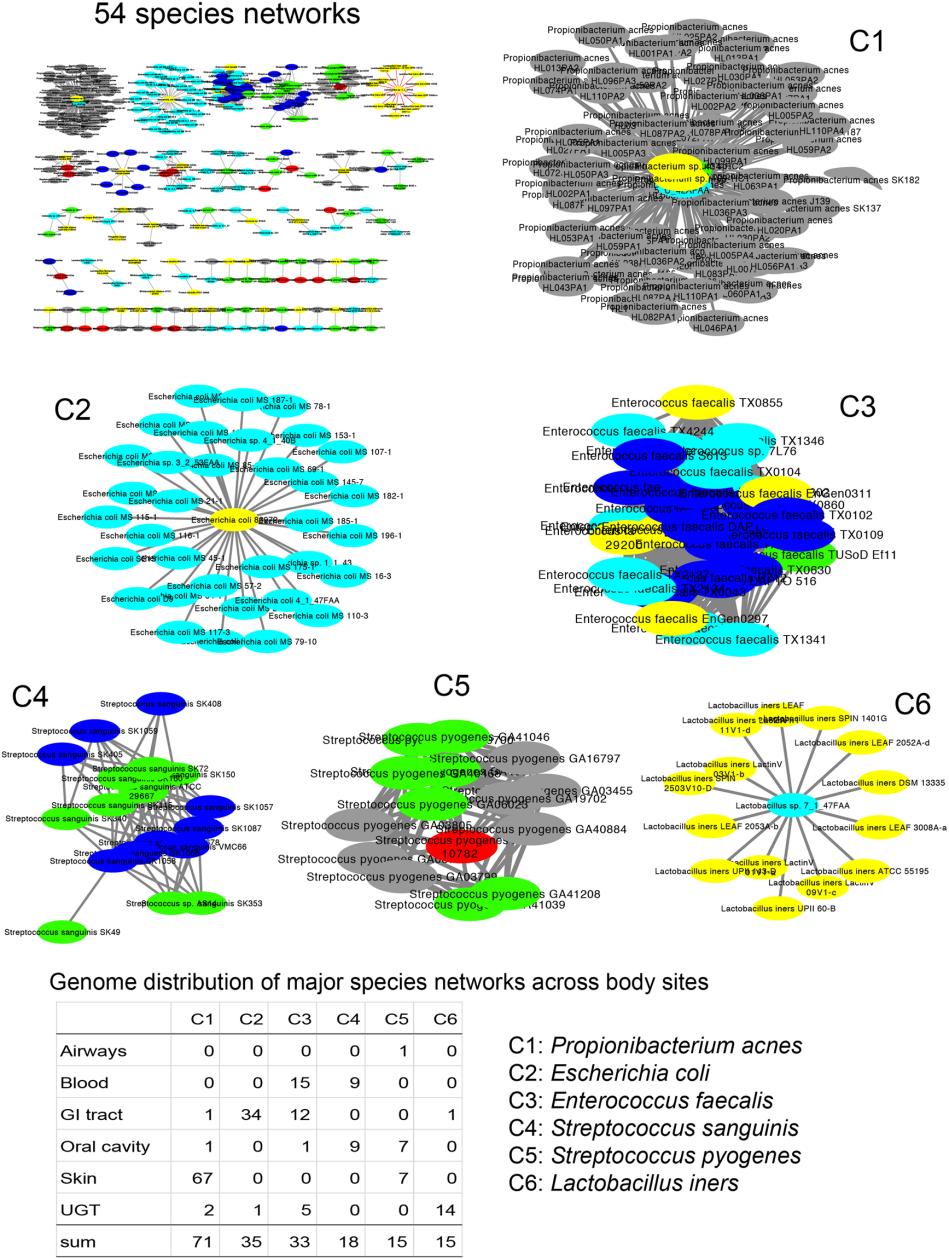
Network visualization of species whose genomes were present in two or more human body sites. A total of 918 genome pairs matched with ANI similarity >95% in different body sites^24^. Data was visualized using Cytoscape ver. 3.6.1^79^. Nodes and edges indicate genomes and links between genomes, respectively. Nodes in red, blue, cyan, green, grey, and yellow represent genomes from airways, blood, GI tract, oral cavity, skin, and urogenital tract, respectively. The visualization resulted in 54 species networks (see the upper left corner), while six major networks (C1 to C6) were magnified for emphasis (consist of >10 genomes, 15 through 71 genomes).

In addition to the phylogenetic effect and the ancient timing of genetic transfers, another possibility to explain the quantitatively greater *inter-niche* vs. *intra-niche* detected HGT events could be direct bacterial DNA transfer from one body site to another either during disease or throughout an individual’s lifespan. Microbiota composition across body sites is known to vary over the timespan of an individual^30,31^. Therefore, we focused on *inter-niche* HGTs involving genomes residing in two different body sites. A total of 15 possible pairwise combinations existed (Table 3, for complete list of 57 possible body site combinations in six body sites see Supplementary Table S7). All five GI tract-related combinations occupied the top six positions in Table 3 highlighting that GI tract microbiota acted as a major “hub” mediating genetic communication and crosstalk with microbiota in other human body sites. The maximum number of *inter-niche* HGTs (*n* = 6,059) occurred between genomes in the GI tract and the oral cavity likely because, (i) the two body sites harbored the maximum number of genomes (452 and 244, respectively), and (ii) the microbiota in the two body sites are anatomically connected (i.e. the oral cavity is the opening to the gastrointestinal tract (e.g. microorganisms in the oral cavity can pass through the intestine and can be detected alongside gut microbiota^30^). Interestingly, however, and despite the blood harboring the least number of genomes, the blood-GI tract *inter-niche* HGTs were the third highest (557) after GI tract-UG tract *inter-niche* HGTs (2,102) (Table 3) suggesting that perhaps genetic information could directly move from the digestive tract to the human circulatory system and later to other body sites. Interestingly, Cystoscape visualization of species multiresidence data (Supplementary Table S4) identified six major networks that connected body sites (Fig. 6). For example, the C3 network (*Enterococcus faecalis*) connected the GI tract, blood and UG tract and the C4 network (*Streptococcus sanguinis*) connected the oral cavity and blood. *E. faecalis* is a common resident of the GI tract but is also known to cause endocarditis and urinary tract infections, which would require entry into the circulatory system^32^. Similarly, *S. sanguinis* is the common resident of the mouth but can reach the bloodstream due to lacerations in the gums. Thus, the bloodstream may serve as conduit for a central role of gut bacteria in influencing other human body sites. However the possible mechanisms mediating such transfers are poorly understood^33^ since bacterial presence in blood is often considered a sign of septicemia or contamination. In particular, it is difficult to imagine how bacterial DNA may pass several layers of tissues to transform resident bacteria in other body sites. Although, circulating cell-free DNA in human bloodstream was first discovered in 1948^34^, its origin, nature, and mechanisms of transfer into the bloodstream remain poorly understood. Therefore, the possible role of bloodstream-mediated HGT across human body sites needs further confirmation.

**Table 3:** Counts (#) of total, *intra-niche*, and *inter-niche* HGT events detected in *mixed* gene sets comprising genomes from only two distinct body sites. For six body sites, a total of 15 possible combinations existed involving only two body sites (for full list of combinations see Supplementary Table S7). Data sorted by the counts of *inter-niche* HGTs in a descending manner. Body sites including the GI tract are highlighted in bold font.

Body site combinations	# HGTs	*# intra-niche* HGTs	*# inter-niche* HGTs
**GI tract, Oral**	**16273**	**10214**	**6059**
**GI tract, UG tract**	**5556**	**3454**	**2102**
**Blood, GI tract**	**2602**	**2045**	**557**
Oral, UG tract	1095	557	538
**GI tract, Skin**	**1006**	**482**	**524**
**Airway, GI tract**	**875**	**477**	**398**
Airway, Oral	491	237	254
Skin, UG tract	322	77	245
Blood, Oral	246	97	149
Oral, Skin	171	48	123
Airway, Skin	75	42	33
Blood, UG tract	29	7	22
Airway, Blood	23	5	18
Blood, Skin	6	0	6
Airway, UG tract	8	3	5

### More than half of the genome in human-associated microorganisms can be influenced by horizontal evolution

**Figure 7:**
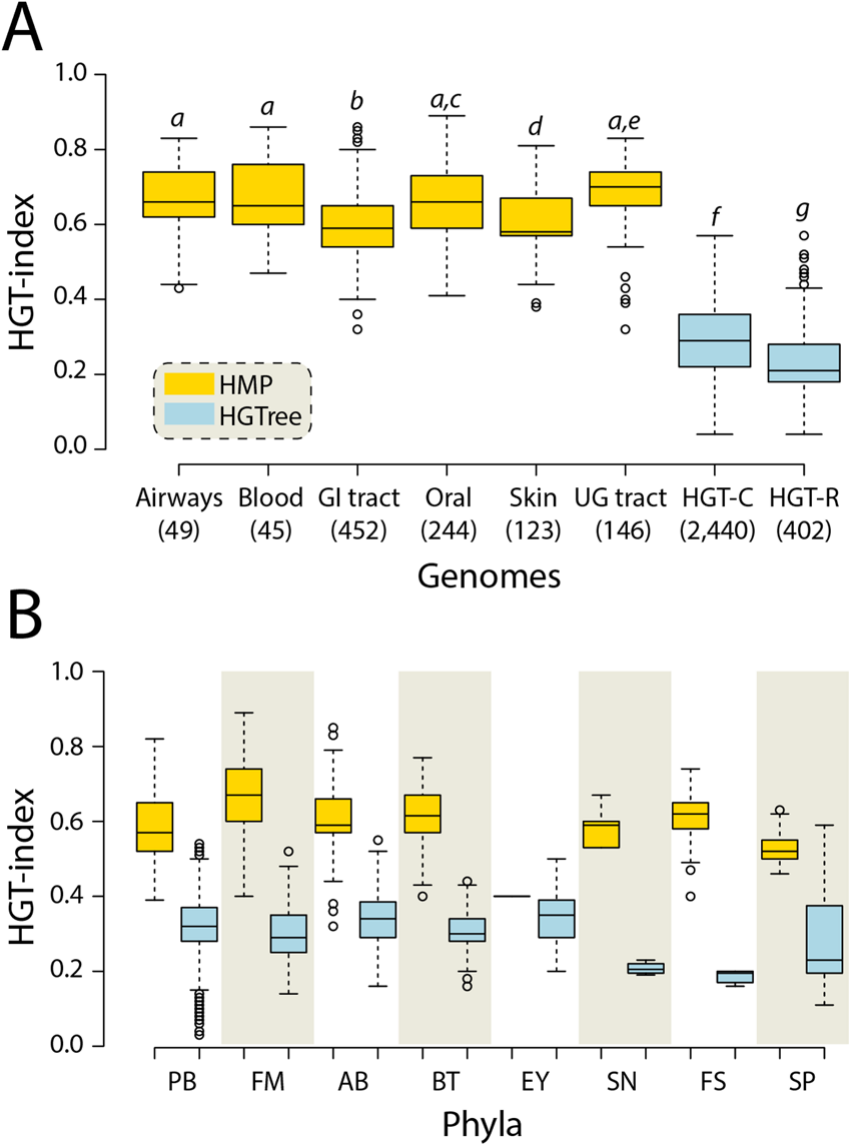
HGT activity increases significantly in human-associated microbes. (**A**) Box plots displaying the distribution of HGT-index for *HMP-genomes* in six body sites, and *HGT-C* (included a total of 2,440 genomes after excluding 32 identical genomes that were part of HMP proteomes) and *HGT-R* (included only 402 proteomes not belonging to any of the 8 HMP phyla) datasets extracted from *HGTree-genomes*. Numbers in parenthesis indicate total number of genomes in each dataset. Statistically significant (Welch’s two-tailed *t*-test with unequal variances, *P* < 0.05) comparisons are indicated in different letters (in italics) on each plot. (**B**) Box plots comparing HGT-index distributions for genomes belonging to phyla common between *HMP*- and *HGTree-genomes*. All comparisons were statistically significant (Welch’s two-tailed *t*-test with unequal variances, *P* < 0.05). PB, Proteobacteria (*n* = 214 *HMP-genomes* vs. 1,037 *HGTree-genomes*); FM, Firmicutes (470 vs. 518); AB, Actinobacteria (197 vs. 243); BT, Bacteroidetes (128 vs. 86); EY, Euryarchaeota (2 vs. 101); SN, Synergistetes (6 vs. 4); FS, Fusobacteria (25 vs. 6); SP, Spirochaetes (17 vs. 43).

Because calculated HGT events are expected to be greater in niches harboring greater number of genomes (e.g. the GI tract), we calculated an HGT-index to normalize the observed HGT-genes by the total number of genes in a genome^16^) (Fig. 7). We compared the HGT-index distribution for each body site against two new datasets customized from *HGTree-genomes*: (i) *HGT-C* including 2,440 genomes after excluding 32 identical genomes with *HMP-genomes*, and (ii) *HGT-R* including only 402 genomes from *HGTree-genomes* not belonging to any of the 8 phyla associated with *HMP-genomes* (highlighted in Supplementary Table S1). Figure 7A displays box plots describing the distribution of the HGT-index in the six HMP-body sites and in the *HGT-C* and *HGT-R* datasets. The global median HGT-index in *HMP-genomes* was 0.63 and was greater than 0.56 in all body sites, indicating that more than half of the genes in the genomes of human-associated microorganisms were exchanged horizontally at some point in evolution. While the number may seem drastically high, these events have accumulated over billions of years of evolutionary time. In fact, a previous study estimated that on average 81 ± 15% genes in the genomes of 181 prokaryotic species had participated in horizontal exchange^29^, which is considerably higher than our estimates.

The HGT-indices for all body sites decreased in the following order: UG tract (0.7), airways and oral cavity (0.66), blood (0.65), GI tract (0.59), and skin (0.57) (Fig. 7A). These numbers were noticeably higher from the median HGT-indices of 0.3 in *HGT-C* and 0.22 in *HGT-R* datasets and from the 0.4 upper-bound we established previously^16^ (*P*-values < 0.05, Welch’s two-tailed *t*-test for unequal variances). In other words, a median HGT reduction of 8% was achieved when microbial phyla known to be associated with the human microbiota were removed from *HGT-C* confirming that HGT activity increases significantly among members of host-microbiota ecosystems^35^. A phylum vs. phylum comparison for 8 phyla common between the *HMP-genomes* and *HGTree-*genomes further confirmed these observations (Fig. 7B, *P*-values < 0.05, Welch’s two-tailed *t*-test for unequal variances). The exercise also supported the initial observation that phylogenetic diversity greatly influences HGT. For example, the gut microbiota, which is the largest and most diverse community of microorganisms among human body sites, had the second lowest HGT-index. Similarly, *HGT-R* and *HGT-C* that include diverse microorganisms from natural habitats (e.g. soil, oceans, lakes, and etc.) had the lowest HGT-indices indicating that greater phylogenetic diversity in environmental samples directly reduces HGT rates.

### The HGT-machinery itself is subject to horizontal evolution

Next, we produced a list of frequently transferred genes (FTGs) by calculating an HGT-index individually for each *HGT-gene* in the *HMP-genomes* dataset^26^. Here, we divided the total number of HGT events detected on each gene tree by the total number of taxa (genomes) present in that gene set to normalize for gene family sizes (Supplementary Table S8 for the top 10% selected *HGT-genes* termed as FTGs). Our FTGs included several genes notorious for their involvement in HGT such as relaxases and bacterial mobilization proteins (MobA/MobL family and MobC; PF03389 and PF05713, respectively), the ‘TraM recognition site of TraD and TraG’ (PF12696) proteins, and the ‘Type IV secretory system Conjugative DNA transfer’ (PF02534), a family of proteins involved in DNA transfer^36^ indicating that HGT machinery itself is subject to frequent transfers, as also shown in ref.^2^. These proteins primarily mediate conjugation, a well-known mechanism of horizontal genetic exchange in bacteria^37,38^. The list also included phage integrase (PF00589, PF13102, and PF02920) and recombinase (PF07508) proteins also indicating the existence of phage-mediated genetic exchange (i.e. transduction) in *HMP-genomes*. However, we also detected type I and III restriction modification DNA specification domains (PF01420, PF04313, and PF04851) and nucleotidyl transferase AbiEii toxin of type IV toxin-antitoxin system (PF08843), which inhibit phage-mediated transfer by acting as an antiviral bacterial defense system^39,40^. Because insertion of foreign DNA into bacterial chromosomes via transduction can (sometimes) be deleterious, it is natural that a counter-evolutionary force would evolve in parallel and be pervasive in horizontal exchange, highlighting the ongoing evolutionary ‘arms race’ confrontation between bacteria and bacterioviruses^41,42^. A gene ontology (GO) enrichment test on FTGs revealed an enrichment of GO terms related to DNA transfer (e.g. DNA recombination, transposition, and integration) and terms involved in metabolic and biosynthetic processes (Table 4, Supplementary Table S9 for complete list). Notably, the most significant GO terms included ‘oxidation-reduction process’ (GO:0055114) and molecular function ‘oxidoreductase activity’ (GO:0016616) consistent with background knowledge on the roles of human gut microbiota in activating host cell signaling through the production of reactive oxygen species in intestinal epithelial cells^43^.

**Table 4:** Significantly enriched biological process GO terms in the top 10% frequently transferred genes (FTGs). Data sorted by the number of GO terms in a descending manner. *FDR*, false discovery rate.

GO ID	#	*P*-value	*FDR*	GO description
GO:0008152	205	2.80E-08	9.20E-07	metabolic process
GO:0044710	132	9.40E-10	9.10E-08	single-organism metabolic process
GO:0006807	107	2.00E-06	2.30E-05	nitrogen compound metabolic process
GO:1901360	84	1.1 E-04	8.4 E-04	organic cyclic compound metabolic process
GO:0046483	82	8.20E-05	7.00E-04	heterocycle metabolic process
GO:0034641	82	3.2 E-04	0.0019	cellular nitrogen compound metabolic process
GO:0006725	81	2.6 E-04	0.0017	cellular aromatic compound metabolic process
GO:0055114	77	8.30E-10	9.10E-08	oxidation-reduction process
GO:0044281	67	9.30E-08	2.60E-06	small molecule metabolic process
GO:1901564	66	3.20E-07	5.10E-06	organonitrogen compound metabolic process
GO:0006139	61	0.0051	0.022	nucleobase-containing compound metabolic process
GO:1901566	47	4.10E-07	5.80E-06	organonitrogen compound biosynthetic process
GO:0016491	44	0.0058	0.046	oxidoreductase activity
GO:0044283	37	1.00E-08	5.00E-07	small molecule biosynthetic process
GO:0044711	37	1.10E-07	2.60E-06	single-organism biosynthetic process
GO:0019752	37	2.10E-05	2.1 E-04	carboxylic acid metabolic process
GO:0043436	37	2.60E-05	2.4 E-04	oxoacid metabolic process
GO:0006082	37	3.10E-05	2.7 E-04	organic acid metabolic process
GO:0006520	34	3.40E-07	5.20E-06	cellular amino acid metabolic process
GO:0044765	31	0.012	0.048	single-organism transport
GO:0006259	30	1.2 E-04	8.4 E-04	DNA metabolic process
GO:0016053	26	6.00E-07	7.30E-06	organic acid biosynthetic process
GO:0046394	26	6.00E-07	7.30E-06	carboxylic acid biosynthetic process
GO:0008652	25	1.00E-08	5.00E-07	cellular amino acid biosynthetic process
GO:1901605	25	2.50E-08	9.20E-07	alpha-amino acid metabolic process
GO:0055086	23	1.00E-04	8.00E-04	nucleobase-containing small molecule metabolic process
GO:0016616	19	2.00E-10	2.20E-08	oxidoreductase activity, acting on the CH-OH group of donors, NAD or NADP as acceptor
GO:0016614	19	1.30E-08	6.10E-07	oxidoreductase activity, acting on CH-OH group of donors
GO:0006812	19	0.012	0.047	cation transport
GO:1901607	17	1.70E-07	3.10E-06	alpha-amino acid biosynthetic process
GO:0006753	17	0.0022	0.011	nucleoside phosphate metabolic process
GO:0016741	17	5.5 E-04	0.012	transferase activity, transferring one-carbon groups
GO:0009117	16	0.0039	0.017	nucleotide metabolic process
GO:0048037	14	0.0014	0.019	cofactor binding
GO:0006732	12	0.0028	0.013	coenzyme metabolic process
GO:0016747	12	0.0014	0.019	transferase activity, transferring acyl groups other than amino-acyl groups
GO:0006790	11	3.00E-04	0.0019	sulfur compound metabolic process
GO:0009110	11	4.5 E-04	0.0025	vitamin biosynthetic process
GO:0042364	11	4.5 E-04	0.0025	water-soluble vitamin biosynthetic process
GO:0006766	11	6.5 E-04	0.0032	vitamin metabolic process
GO:0006767	11	6.5 E-04	0.0032	water-soluble vitamin metabolic process
GO:0016835	11	0.0035	0.034	carbon-oxygen lyase activity
GO:0004803	10	1.70E-08	6.10E-07	transposase activity
GO:0032196	10	1.60E-07	3.10E-06	transposition
GO:0006313	10	1.60E-07	3.10E-06	transposition, DNA-mediated
GO:0006310	10	3.50E-06	3.80E-05	DNA recombination
GO:0044272	10	9.90E-05	8.00E-04	sulfur compound biosynthetic process
GO:0009108	10	0.0025	0.012	coenzyme biosynthetic process
GO:0050662	10	0.0018	0.022	coenzyme binding
GO:0016836	9	6.8 E-04	0.012	hydro-lyase activity
GO:0009066	8	1.30E-05	1.3 E-04	aspartate family amino acid metabolic process
GO:0072527	8	4.4 E-04	0.0025	pyrimidine-containing compound metabolic process
GO:0006575	8	0.0071	0.029	cellular modified amino acid metabolic process
GO:0072528	7	5.4 E-04	0.0028	pyrimidine-containing compound biosynthetic process
GO:0009401	7	5.4 E-04	0.0028	phosphoenolpyruvate-dependent sugar phosphotransferase system
GO:0016407	7	1.1 E-04	0.0031	acetyltransferase activity
GO:0008643	7	0.0037	0.016	carbohydrate transport
GO:0042398	7	0.0037	0.016	cellular modified amino acid biosynthetic process
GO:0008509	7	0.0041	0.035	anion transmembrane transporter activity
GO:0015074	6	1.2 E-04	8.4 E-04	DNA integration
GO:0009067	6	1.2 E-04	8.4 E-04	aspartate family amino acid biosynthetic process
GO:0016410	5	0.002	0.022	N-acyltransferase activity
GO:0009072	5	0.007	0.029	aromatic amino acid family metabolic process
GO:0016645	5	0.0037	0.034	oxidoreductase activity, acting on the CH-NH group of donors
GO:0015291	5	0.0065	0.047	secondary active transmembrane transporter activity

### Recently transferred genes (RTGs) are poorly annotated and understood

Earlier we suggested that *one-to-one* gene transfers were more likely to have occurred relatively recently in evolution. These transfers could yield insights into the nature of modern-day genetic exchange mediated by microorganisms associated with the human body. Therefore, we focused on genes with relatively higher proportions of *one-to-one* recent HGT events to produce a list of RTGs (Supplementary Table S10). Notably, the list included several proteins of unknown functions (indicating either poor annotation of *HMP-genomes* or an abundance of novel protein families for which little is currently known), and viral proteins and transposases, in addition to proteins involved in transcription regulation (Supplementary Table S10). For example, the ‘Superinfection exclusion protein B’ (PF14163) is a family of bacterial proteins that fights super-infection phages insensitive to repression. For this protein, 5 out of 6 detected HGTs were *one-to-one*. Similarly, the list included the ‘Biofilm development protein YmgB/AriR’ (PF10798), a family involved in biofilm formation and acid resistance, where 8 out of 12 HGT events were *one-to-one*. The enriched GO terms included several biological processes related to biosynthesis and metabolism, transport, and regulation, consistent with the modern understanding of human microbiota roles in metabolism and food digestion^44^ (Supplementary Table S11).

### *HGT-free* genes can guide robust reconstructions of phylogenies describing the history of life

We also identified 191 genes tentatively termed *HGT-free* genes (filtered from a total of 26,298 genes using a criterion of presence in at least 10 genomes) since they produced no detectable conflict evidence of HGT during reconciliation with corresponding 16S rRNA species trees. (Supplementary Table S12). The list included the DNA methylase protein (69 genomes in 5 genera) that plays important roles in cellular defense against exogenous DNA, cell replication, sequence mismatch correction, and gene expression regulation^45^. The GO enrichment test indicated that the cellular component GO term ‘bacterial-type flagellum’ [GO: 0009288] was enriched in *HGT-free* genes (Supplementary Table S12). This result is consistent with a previous study revealing that phylogenetic trees built from concatenation of 14 core sets of flagellar genes were highly consistent with corresponding species trees^46^. Therefore, one utility of *HGT-free* genes could be in the reconstruction of phylogenetic trees describing the history of (prokaryotic) life since identification of vertically inherited marker genes is vital to producing error-free phylogenies. *HGT-free* genes however tended to be less widespread among *HMP-genomes* (e.g. 69 genomes for ‘DNA methylase’ protein family being the highest representation, Supplementary Table S12) thus diminishing their utility as orthologous gene markers in broad phylogenetic studies. This prompted us to identify ‘widespread’ genes in *HMP-genomes* and to evaluate their sensitivity to HGT.

**Figure 8:**
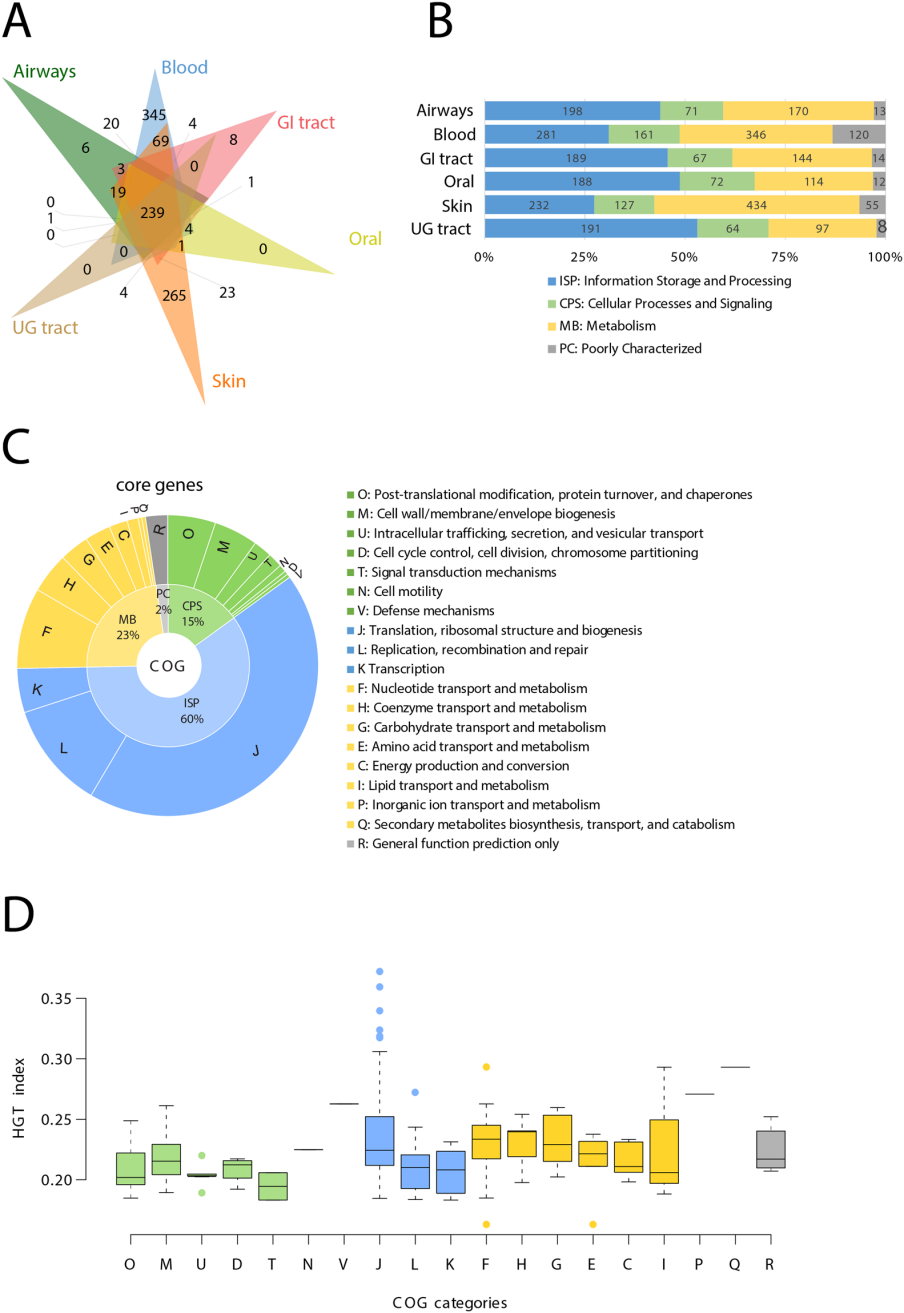
Widespread and core genes in human microbiota. (**A**) Venn diagram highlights the distribution of widespread genes in each body site. Widespread genes defined by genes present in >70% of genomes of that body site or its combinations. (**B**) Bar plots illustrate the proportion of COG functional categories mapped to total widespread genes in each body site. (**C**) Pie chart indicate the enrichment of COG functional categories in core genes that by definition were widespread in all six body sites. (**D**) Box plots compare HGT-index distributions of core genes, as distinguished by COG categories. HGT-index is the number of HGT events detected on a gene tree divided by the total number of taxa in that gene tree.

Widespread genes were defined by genes present in >70% of genomes of a body site or its combinations with the other body sites (Fig. 8A). The 6-way Venn diagram illustrates the number of widespread genes that were either unique to each body site or were shared by them (Fig. 8A). There were no widespread genes unique to either oral cavity or the UG tract likely because both body sites are highly diverse in their composition of genera and species (Fig. 2). For example, the 244 microbial genomes in the oral cavity belong to 64 different genera (the second largest after the gut microbial community, Table 2). Similarly, the 146 microbial genomes in the UG tract belong to a total of 41 distinct genera, the next largest among body sites (Table 2, Fig. 2). Therefore, the oral cavity and the UG tract appeared highly heterogeneous in the composition of human microbiota genera. In turn, only 6 and 8 genes were uniquely widespread in the airways and the GI tract, respectively (Fig. 8A). The gut microbiota is the largest (452 genomes) and the most diverse (99 genera and 271 species) ecosystem in the human body (Table 2, Fig. 2). Thus, it is unsurprising that very few genes were uniquely widespread among members of the gut microbial community consistent with its diverse metabolic and physiological roles in the human body^43^. Surprisingly, however, blood and skin microbiota encoded 345 and 265 widespread genes, despite harboring only 45 and 123 genomes, respectively (Fig. 8A). Indeed, the two body sites were also relatively less diverse (8 and 16 distinct genera, Table 2, Fig. 2) indicating that species composition in blood and skin was relatively more homogenous within the body site than among body sites. Finally, a total of 239 genes were widespread when all genomes from six body sites were considered as a whole and were termed ‘core’ genes. Next, we matched all widespread genes in every body site to the clusters of orthologous groups (COG)^47^ general categories of molecular functions: ‘Information Storage and Processing’ (ISP), ‘Cellular Processes and Signaling’ (CPS), ‘Metabolism’ (MB), and ‘Poorly Characterized’ (PC) (Fig. 8B). The majority of the widespread genes in all body sites were preferentially involved in ISP and MB functions (Fig. 8B). A mapping of core genes to detailed COG categories confirmed that 43.5% of core genes were annotated to ‘Translation, ribosomal structure and biogenesis’, followed by ‘Replication, recombination and repair’ (11.5%), and ‘Nucleotide transport and metabolism’ (8.7%), corresponding to ISP and MB (Fig. 8C, Supplementary Table S13).

One utility of core genes can be in large-scale phylogenies where universal genes are often concatenated in attempts to resolve the history of life. These concatenations often involve ribosomal proteins^48,49^ and are now generally preferred over single-gene phylogenetic analyses due to increased resolution despite concerns that concatenated proteins may have independent evolutionary histories^50–53^. Interestingly, an HGT-index distribution of core genes revealed that genes involved in metabolic and information functions tended to have higher HGT-indices than CPS genes (Fig. 8D). Surprisingly, the outliers with extreme HGT-indices included mostly ribosomal proteins (28 out of top 50 genes, Supplementary Table S13) in addition to translation initiation factor IF-1 (TIF1, COG0361, the highest HGT-index of 0.37 for 383 detected HGT events in a gene tree containing 1,030 taxa), and other popular phylogenetic markers such as translation elongation factors (Supplementary Table S13). Therefore, an HGT analysis of core genes questions the reliability of utilizing ribosomal proteins in concatenated phylogenetic analyses aimed towards uncovering the origin of life, a central question in evolutionary biology research, and demand caution and detailed evaluation^53,54^. Based on our preliminary exploration, we produced a list of 31 core genes present in >90% *HMP-genomes* with HGT-index <0.2 as potential candidates for future studies focused towards exploring deep evolutionary relationships among microbial lineages (Supplementary Table S14). These genes could become reliable candidates for concatenation and reconstruction of more reliable phylogenies that are less sensitive to non-vertical evolution and could provide more resolution than the 16S rRNA tree (read below).

## Discussion

We performed an in-depth analysis of HGT activity and sensitivity in the genomes of human-associated microorganisms. We utilized an explicit evolutionary method of HGT detection based on phylogenetic tree reconstructions and reconciliations to detect HGT^16^. Specifically, we reconstructed >80,000 gene trees and reconciled each and every gene tree against its corresponding reference (or species) tree built from the 16S rRNA gene alignment. Tree conflicts arising due to HGT (evaluated under a parsimony framework^20^) were analyzed against a control dataset of prokaryotic genomes sequenced from diverse environments including human microbiota^16^.

Overall, we observed significantly higher HGT activity occurring in the human-associated microorganisms relative to the control group both in terms of the number of HGT genes and the number of HGT events per gene, which is confirmatory of previous findings^2^. Observed gene transfers were classified into *intra-* and *inter-niche* gene transfers occurring among microorganisms sharing the same body site and dispersed across body sites, respectively. We observed that *intra-niche* gene transfers comprised roughly 40% of the total HGT activity. In general, microorganisms sharing the same niche are united by ecology, spatial proximity, and also phylogenetic similarity since closely-related microorganisms are expected to colonize and adapt to similar habitats. All of these factors can increase HGT activity among microorganisms sharing the same niche.

In turn, *inter-niche* gene transfers that occur among microorganisms occupying distant and different body sites pose questions regarding their mechanics and timings. We present two explanations for the quantitatively greater number of *inter-niche* HGTs. First, such transfers could predate human colonization of microbial organisms. If true, such transfers should be more widespread in nature (since they had more time to accumulate among genomes) and involve distantly related and many prokaryotic species. Indeed, HGT-ratios of *many-to-many* and *one-to-many* gene transfers were highest for phylogenetically diverse microorganisms occupying diverse habitats (Figs. 4B and 4C). In turn, HGT-ratios for *one-to-one* transfers were highest for phylogenetically similar microorganisms occupying similar or different habitats (Fig. 4A), which supported a tendency for microorganisms to preferentially exchange genes with closely-related microorganisms regardless of spatial proximity. Median protein sequence identity for *one-to-many* and *many-to-many* events was also lower compared to *one-to-one* events (Fig. 5) further supporting the idea that *one-to-many* and *many-to-many* HGTs were likely more ancient than *one-to-one* transfers. A second intriguing possibility could be the direct movement of bacterial DNA or bacteria from the gut to other body sites via the bloodstream to transform bacterial cells residing in other body sites. However, the mechanisms responsible for such transfers are presently poorly understood and demand further exploration. Since the microbiota composition across body sites can change throughout an individual’s lifespan^30,31^, it is also possible that some microorganisms currently assigned to one body site, for example, the GI tract, were found in the oral cavity at an earlier time in the individual’s lifespan and interacted and exchanged genes.

We are confident that *inter-niche* HGTs are not heavily biased by species or strain multi-residence across human body sites. None of the analyzed genomes were duplicated across body sites at strain level and very few species (6.3%) were present in more than one body site (Fig. 2). As per ANI binning of species^24^, only 918 out of 408,385 genome pairs matched with >95% identity across different body sites, which amounts to <1% species-level multi-residence (Supplementary Table S4). Notably, the 918 multi-residence ANI matches corresponded to important bacteria that are known to transcend from their primary body site (gut and oral cavity) and reach other body sites (e.g. UG tract) perhaps via the bloodstream (Fig. 6). It can also be argued that some hitherto not-sequenced genomes from bacterial species may be discovered later from multiple body sites and bias our *inter-niche* HGT estimates. However, this issue applies to all genomic datasets publicly available from NCBI and other common platforms since spatial coverage for the majority of microorganisms on Earth effectively remains unknown. Continuous sequencing efforts will no doubt help bridge the gap but this limitation is often beyond the control of bioinformaticians and data scientists who rest their conclusions on presently available data.

We used the HGT-index as proxy to describe the tendency of genomes and individual genes to participate in HGT (see refs.^16,26^ for previous applications of the HGT-index). The meaning of the HGT-index in genomes is straightforward - it is the fraction of total genes that participate in HGT. The HGT-index for genomes can thus be influenced by the inclusion of low-quality or poorly-annotated genomes for which many ORFan genes will miss inclusion in the putatively orthologous gene sets. In turn, the HGT-index for genes reports the number of detected HGT events on that gene tree divided by the total number of genome (taxa) members of that gene tree. Gene trees could be highly heterogeneous in taxa composition as some genes could have broad distribution across many genera and phyla while others would be narrowly distributed. Even in the highly heterogeneous gene trees, HGT events could still be restricted to only a particular small group of microorganisms. Accounting for these factors in HGT-index calculations is a work in progress^26^. In the present study we normalized HGT events by total taxa since the true potential of HGT participation of any gene should take into account both genomes that do and do not exchange that gene horizontally (see Discussion in ref.^26^). These limitations of the HGT-index should be considered when evaluating our results.

We compared the HGT-indices of *HMP-genomes* against the pre-calculated HGT-indices in *HGTree-genomes*. Both datasets were generated in a similar manner except for two notable differences. First, orthologous gene sets in *HMP-genomes* were produced by ProteinOrtho^55^ that is a faster alternative to MestOrtho^56^. The two orthology detection software however identified proportionally the same number of orthologous gene sets (e.g. ~89,000 gene sets in 1,059 *HMP-genomes* vs. 154,000 gene sets in 2,472 *HGTree-genomes*) and hence are not expected to bias our results, especially because the downstream HGT detection strategy using tree reconciliation was similar in both HMP and *HGTree* datasets. Second, we also performed HGT analysis on large gene trees in *HMP-genomes* containing >50% genomes that were excluded from *HGTree-genomes* because they can also be subject to horizontal evolution. However, large gene sets only constituted 0.77% (634/81,357) of total gene sets and thus are not expected to numerically bias the comparisons. Just like the HMP dataset, the HGTree dataset also likely includes genomes occupying multiple residences, which are not completely known. This is a knowledge gap that needs to be filled.

Our study is restricted to only HGT-derived genes in “normal” hosts, as defined and included by the HMP in their study^18^. Thus, the potential role of microbiota-mediated HGT in human disease is not depicted by our study. These tasks can however be easily accomplished using the online *HGTree* webserver^16^ that provides user-friendly tools for large-scale HGT evaluation of user-provided genomic datasets. We chose to work with the HMP dataset because it is one of the “gold-standards” in microbiome research and is widely used by the scientific community. Recall that studied HMP reference genomes were sequenced from different individuals. Therefore, our analysis can miss the very recent HGT events that have occurred during the life course of each individual. A recent study has expanded the HMP dataset and attempted to provide a baseline microbiome composition across individuals over multiple time points^57^. Such efforts will ensure that we have, in the future, sufficient reference genome data from different body sites in the same individuals.

Finally, three major limitations/challenges of the phylogeny-based HGT detection method should be noted. First, it can fail to detect HGT occurring between closely-related strains belonging to the same species as they do not produce species tree conflict (note that we excluded gene sets harboring genomes belonging to only one species from the analysis, see Methods). In such cases, composition-based HGT detection methods (see Liu *et al*.^3^ for detection of HGT events in 308 human-associated microbial genomes using composition-based biases) might be more valuable. However, composition-based methods tend to give contrasting results when the methods are changed (see ref.^14^ for a benchmark study on 16 composition-based methods) and cannot reliably detect ancient HGT events (see refs^12,13^ for challenges). Hence, there is a definite need to couple multiple approaches of HGT detection for improved global precision in HGT estimation.

Second, an important aspect is the reconciliation of gene and species trees. Multiple reconciliations may exist that may be optimal (i.e. most parsimonious). Therefore, it is important to traverse through the entire solution search space and to produce quantitative indicators (similar to bootstrap support for trees) to support selected reconciliations^58^, a task that is computationally intensive. Bansal *et al*.^58^ however confirmed that the majority of the gene family evolution events (duplication, transfer, and loss) remain conserved among most parsimonious reconciliations indicating that HGT assignment based on random selection among multiple optimal reconciliations should be considered reliable. Similarly, HGT detection via tree reconciliation can differ according to the different event costs, choice of alternatively rooted gene trees among equally optimal rootings, the existence of multifurcated (non-binary) branches, and inability to handle species tree with poorly supported nodes. These limitations that imply incompleteness of the DTL model^20^ cannot be overcome since searching for the entire solution space is computationally unfeasible, especially considering the size of our datasets.

Third, an important dilemma is whether to build phylogenetic trees using single-gene (this study) or concatenated genes (e.g. refs^48,59^). The latter has become popular because it provides more resolution than single-genes. However, concatenated gene sets pose additional problems because member genes could have independent evolutionary histories^60,61^ (as also demonstrated by the high HGT-indices of ribosomal proteins in Supplementary Table S13 that are popular markers in gene concatenation), in addition to gaps introduced by heterogeneous protein domain make-up among distantly-related taxa^51^. Since we rooted prokaryotic trees using the eukaryotic outgroup sequence (i.e. 18S rRNA from *Saccharomyces cerevisiae*), it is also difficult to establish *a priori* how many of the bacterial single-copy genes (e.g. 120 from ref.^59^) will have homologs in *S. cerevisiae* for downstream phylogenetic analysis. In practice, the number of universal single-copy genes truly conserved across Bacteria and Eukarya declines sharply with the increase and diversity in genomes being studied^62^. Nevertheless, when there is a consistent signal of HGT between donor and recipient species, it will no doubt prove more useful to concatenate those genes into a single alignment to improve resolution. It will therefore be important to identify single-copy genes conserved across a wide range of organisms that are mostly inherited vertically to hopefully improve species tree resolution. In this regard, our identification of the 31 core genes with HGT-index <0.2 and distributed in >90% of *HMP-genomes* may offer an interesting starting point (Supplementary Table S14). We would like to carefully study this possibility in the future and highlight here that the 16S rRNA tree does not fully resolve the bacterial tree of life but it is the best method given the challenges mentioned above.

In sum, we expect that the phylogeny-based HGT detection method presented in this study will facilitate large-scale simultaneous analysis of (meta)-genomes routinely produced by sequencing platforms and will aid in our understanding of the many complex interactions of humans with the microbial inhabitants of the planet. The method may especially be superior for detection of ancient HGT events but poses several technical and conceptual challenges that we have also attempted to address or highlight in this study. The open challenges are to adapt the phylogeny-based HGT detection pipeline to also integrate viral and eukaryotic genomes since viruses are now recognized as major players in gene transfer and innovation^63–65^ and eukaryotes are also subjected to gene transfer (see refs.^66,67^ for debate). Both genomic datasets however pose unique challenges since viral genes are highly variable and lack a conserved marker like the 16S rRNA gene to produce reliable phylogenies. In this regard, utilizing structure-based approaches may be more fruitful^68,69^. In turn, eukaryotic genomes are several times larger than prokaryotic genomes and include many non-coding regions that can also be inherited horizontally. We hope to incorporate these solutions into a future release.

## Methods

### HMP reference genome data retrieval and manipulation

A total of 1,304 non-redundant (strain-level) prokaryotic genomes were downloaded from the HMP Data Analysis and Coordination Center (DACC) (last updated: October 10, 2014, download date: November 2015)^70^. These genomes corresponded to the following human body sites: airways (no. of genomes = 49), blood (45), GI tract (452), oral (244), skin (123), urogenital (UG) tract (146), heart (2), liver (1), lymph node (1) and unknown (240). Heart, liver, and lymph node were subsequently removed from the analysis, as they did not meet the four-taxa minimum requirement needed to reconstruct a phylogenetic tree. Organisms with unknown body affiliations were also excluded. This reduced the dataset to a total of 1,059 non-redundant prokaryotic genomes including 1,057 bacteria and 2 archaea (both in the GI tract) corresponding to six major body sites (airways, blood, GI tract, oral, skin, and UG tract) and comprising of 7 bacterial (Actinobacteria, Bacteroidetes, Firmicutes, Fusobacteria, Proteobacteria, Spirochaetes, and Synergistetes) and 1 archaeal phyla (Euryarchaeota), 152 genera and 591 distinct species (Supplementary Table S2 and Fig. 2).

### Identification of multi-resident and contaminant species and strains

The possibility that any *HMP-genome* resides in two or more human body sites was examined at both strain and species levels. For this, we utilized average nucleotide identity (ANI) that is the standard measure to demark microorganisms using genomic sequences^24^. Using the fast implementation of FastANI, the ANI similarity (%) of genome pairs was calculated with default options. Since we were interested in the multi-residence of a genome, genome pairs derived from the same body sites were excluded in this analysis and pairs of genomes that belonged to different genera were also not compared since they cannot be grouped together into species. Exceptionally, self-to-self comparisons of each of the 1,059 *HMP-genomes* was conducted as a positive control to check the reliability of the ANI value calculation. After calculating the ANI values of all possible genome pairs, thresholds 99.9% and 95% were applied to bin genomes at strain and species level, respectively. For example, genomes having identity >95% ANI threshold and derived from different body sites were regarded as multi-residents at the species level. In addition, the possibility that some of the 1,059 HMP-genomes were derived from non-human sources was also evaluated due to possibility of contamination arising during DNA extraction and sequencing library preparation. Salter *et al*.^23^ previously identified 93 candidate genera commonly contaminated in clinical genomic or metagenomic samples. Thus, scientific names of *HMP-genomes* were compared with contaminant genera names to identify suspected contaminants.

### Retrieval of pre-calculated HGT events from the *HGTree* database

Pre-calculated HGT events in a total of 2,472 non-redundant (strain-level) prokaryotic genomes (156 archaea and 2,316 bacteria) corresponding to 41 phyla, 640 genera, and 1,069 species were retrieved from the *HGTree* database (the *HGTree-genomes*) (http://hgtree.snu.ac.kr/)^16^. Two new datasets were customized: (i) HGTree-Complete (*HGTree-C*) after removing 32 identical (strain level) genomes between the *HMP-genomes* and the *HGTree-genomes*, and (ii) HGTree-Reduced (*HGTree-R*) after removing 2,070 genomes in *HGTree-genomes* belonging to any of the 8 HMP phyla (Supplementary Table S1).

### HGT detection in *HMP-genomes*

The HGT detection strategy largely followed the methods described in ref.^16^ except for two notable differences: (i) ProteinOrtho (ver. 5.15)^55^ was used instead of MestOrtho^56^ for generation of “putative” orthologous gene sets due to its greater efficiency in handling large datasets (gene sets are termed putative orthologs because we test for their participation in HGT during downstream analysis), and (ii) gene sets comprising >50% genomes were removed from downstream analysis in^16^, as they are more likely to evolve vertically^71^ and their processing was computationally intensive. Due to the fast speed of ProteinOrtho, and because some horizontally transmitted genes could be widespread among extant species, we chose to keep the large gene sets comprising >50% genomes in the present analysis (only contributed 0.77% additional gene sets). However, gene sets that included genomes of only one species (yield low resolution in reference species trees) or contained <4 genomes (a requirement of building phylogenetic tree) were removed from the analysis, similar to ref.^16^ along with other details. Briefly, 16S rRNA genes were called by RNAmmer (ver. 1.22)^72^. Orthologous genes for each species were mapped to corresponding 16S rRNA genes. CLUSTAL-Omega (ver. 1.2.1)^73^ was used for aligning orthologous and 16S rRNA gene sets. A profile alignment of 16S rRNA genes was generated combining with the yeast 18S rRNA sequence from *S. cerevisiae*, which was used as an outgroup to root the species tree and then removed during tree reconciliation step. Gene sets where all pair-wise neighbor-joining (NJ) distances were close to zero were filtered to improve resolution during downstream steps of tree reconciliation. FastTree (ver. 2.0) was used for generation of “approximate” ML gene and species trees using the default JTT + CAT amino acid substitution model^22^. Phylogenetic splits reliability was measured by the ‘local support values’ based on Shimodaira-Hasegawa (SH) test^74^. RANGER-DTL-U (ver. 1.0) with default parameters (-D 2 -T 3 -L 1) was used to reconcile each gene tree against its corresponding reference species tree (see ref.^20^ for details). Specifically, the program embeds each gene tree onto its corresponding species tree by mapping each node of the gene tree onto a unique node of the species tree and assigning one of the four possible evolutionary events (i.e. speciation, duplication, transfer, or loss) to nodes on the gene tree. Total cost of embedding (reconciliation) is calculated in terms of assigned values of DTL parameters. Multiple embeddings are possible for each gene tree inside the species tree and the embedding where total cost is the minimum is considered the most optimal reconciliation^20^. Multiple optimal solutions may exist, however, event assignments tend to generally remain conserved for the most part^58^. For nodes representing transfers, the program also identifies the edge on the species tree that constitutes the edges of the gene tree node to label the recipient species of transfers^20^. Because gene trees were unrooted and species trees were rooted, reconciliation was done by considering all possible rootings for gene trees followed by random selection of a root amongst rootings that yielded the most parsimonious reconciliation. For each gene and corresponding species tree, local support values based on SH test^74^ were calculated along with direction of gene transfer (i.e. designation of both the donor and recipient genomes) and labeling of the “candidate” HGT events. HGT-participating genes (hereafter *HGT-genes*) were assigned to microbial genomes residing in six body sites.

### Protein family assignments and functional annotations

PfamScan (ver. 1.3)^75^ was used for assignment of protein families (PFs) to a protein of median length selected from each gene set. To test the reliability of this approach, we scanned all proteins in 500 randomly selected gene sets against the Pfam library. A PF was assigned to a gene set when >50% of its member proteins matched to the same PF (i.e. 50% majority rule). We discovered that our approach (i.e. scanning a protein of median length) versus the majority rule yielded the identical PF in 493/500 gene sets (i.e. 98.6% overlap). Due to the sheer size of our datasets (>80,000 gene sets alone in *HMP-genomes*), we therefore preferred to assign PFs to proteins of median length selected from each gene set rather than scanning all proteins in every gene set against the entire Pfam database with understanding that this approach will have <2% error that was, however, greatly offset by a significant reduction in computing time (e.g. it took ~17 hours to process 500 gene sets on a typical Desktop computer). GO enrichment using hypergeometric test was conducted using an open-source R package of the domain-centric gene ontology (dcGO) resource (ver. 1.05)^76^. PF Ids were provided as input (*foreground*) and PFs corresponding to the prokaryotes-specific subset of GO terms (*n* = 8,042) taken from AmiGO2 ver. 2.4^77^ (download date: November 2016) were provided as *background*.

### HGT-index and classification of ‘frequently’ and ‘recently’ transferred proteins

An HGT-index was defined for each genome and every gene set. For genomes, the index represents the total number of genes that participate in HGT divided by the total number of genes in that genome^16^. For genes, the index represents the total number of HGT events detected in that gene tree (when reconciled with the corresponding reference species tree) divided by the total number of genomes (taxa) member of that gene tree^26^. Both indices are given on a scale from 0 to 1 with higher values representing higher tendencies to participate in HGT. We defined ‘frequently transferred genes’ (FTGs) as genes in the top 10% HGT-index distribution for all genes. We defined ‘recently transferred genes’ (RTGs) as genes where HGT events occurred directly between species in a *one-to-one* manner (Fig. 2). In order to minimize small sample size related biases, we removed gene sets that contained either <5 HGT events, <5 distinct genera, or <10 distinct genomes.

### HGT analysis of widespread and core genes

Widespread genes were defined as genes present in >70% of genomes of a body site (e.g. the GI tract) or its combinations with other body sites (e.g. microbial genomes of the oral cavity and skin). Widespread genes present in all six body sites were termed core genes. These proteins were mapped to COG categories by scanning all proteins in the gene set against the COG database (ftp://ftp.ncbi.nih.gov/pub/COG/COG2014/data/)^47^ using BLASTP (as above). COGs were annotated using the 50% majority rule, as in^78^.

### Network analysis

Sequence similarity between genomes derived from different body sites was calculated using the ANI method^24^ as mentioned above. Genome pairs with >95% ANI similarity (threshold for demarcating prokaryotic species) were chosen and imported into Cytoscape ver. 3.6.1 to be integrated into species networks^79^.

## Supplementary information

Supplementary Materials

Table S1

Table S2

Table S3

Table S4

Table S5

Table S6

Table S7

Table S8

Table S9

Table S10

Table S11

Table S12

Table S13

Table S14

## Data Availability

Multiple sequence alignments and maximum likelihood phylogenetic trees generated from *HMP-genomes* can be retrieved from https://figshare.com/s/4bdbb083ff00d2ffebd1. Similar data for *HGTree-genomes* is already available from the HGTree website (http://hgtree.snu.ac.kr/) under Downloads. Users can reuse data for academic use with an acknowledgement and citation to the present study.
