# Supplementary material for: Horizontal gene transfer in human-associated microorganisms inferred by phylogenetic reconstruction and reconciliation
Source: Sci Rep. 2019 Apr 11;9:5953. doi: 10.1038/s41598-019-42227-5 (PMC6459891; doi:10.1038/s41598-019-42227-5)
Supplement: Supplementary file 1 — Supplementary Materials [file 41598_2019_42227_MOESM1_ESM.pdf]

Horizontal gene transfer in human-associated microorganisms inferred by phylogenetic reconstruction and reconciliation

Hyeonsoo Jeong[1,2], Bushra Arif[3], Gustavo Caetano-Anollés[4], Kyung Mo Kim[5*], Arshan Nasir[3*]

[1]Department of Animal Sciences, University of Illinois at Urbana-Champaign, Urbana, IL, USA.
[2]School of Biological Sciences, Georgia Institute of Technology, Atlanta, GA, United States
[3]Department of Biosciences, COMSATS University Islamabad, Park Road, Tarlai Kalan, Islamabad, Pakistan
[4]Department of Crop Sciences, University of Illinois at Urbana-Champaign, Urbana, IL, USA
[5]Division of Polar Life Sciences, Korea Polar Research Institute, Incheon, Republic of Korea

[*]For correspondence: arshan.nasir@gmail.com and kmkim@kopri.re.kr

Supplementary Materials

Table S1. *HGTree-genomes* analyzed in this study. HGT-index is the number of HGT-genes in a genome divided by the total number of genes in that genome. Phyla common between *HGTree-genomes* and *HMP-genomes* are highlighted.

Table S2. *HMP-genomes* analyzed in this study. HGT-index is the number of HGT-genes in a genome divided by the total number of genes in that genome.

Table S3. ANI multi-residence test on strains. Highlighted rows indicate probable multiresidence cases.

Table S4. ANI multi-residence test on species.

Table S5. Number of genera matching the list of possibly contaminant genera.

Table S6. Pairwise Mann–Whitney *U* test to evaluate statistically significant comparisons among phylogenetically similar and diverse microorganisms occupying similar and diverse habitats. Bonferroni adjusted *P*-values are listed, where available.

Table S7. Number of total, *intra-niche*, and *inter-niche* detected HGT events in all body site combinations.

Table S8. Description of top 10% frequently transferred genes (FTGs). Top 10% determined by HGT-index distribution, which, in the case of individual genes/proteins, is the number of detected HGT events on a gene tree divided by total number of genomes (taxa) member of that gene tree. Ortholog Id is a unique identifier assigned to each putative orthologous gene set produced by ProteinOrtho[64]. Matching PFs and GOs are also listed next to each FTP. BP, biological process; CC, cellular component; MF, molecular function, as defined by the GO hierarchy.

Table S9. GO terms significantly enriched in the top 10% frequently transferred proteins, as identified by their HGT-index value. PFs matching to GO terms are also listed.

Table S10. Description of recently transferred genes (RTGs) that were not identified as FTPs in the *HGTree-genomes*. PF and GO Ids and descriptions are listed next to each RTP. HGT-index is the number of *one-to-one* HGT events divided by the total number of detected HGT events on that gene tree.

Table S11. GO terms significantly enriched in the top 10% recently transferred genes, as identified by their HGT-index value. PFs matching to GO terms are also listed.

Table S12. Description of *HGT-free* genes. *HGT-free* genes did not produce detectable tree conflict during reconciliation of gene and species trees. Only genes present in at least 10 genomes are listed.

Table S13. HGT potential of core genes. Core genes were present in >70% of total sampled *HMP-genomes* and detected in each of the six body sites studied. Ortholog Id is a unique identifier assigned to each putative orthologous gene set produced by ProteinOrtho. Matching COG categories are also described. HGT-index is the number of detected HGT events on that gene tree divided by the total number of taxa. Ribosomal proteins are highlighted.

Table S14. List of 31 genes detected in more than 90% *HMP-genomes* with HGT-index < 0.2.
